# Beneficial Effects of Autologous Bone Marrow-Derived Mesenchymal Stem Cells in Naturally Occurring Tendinopathy

**DOI:** 10.1371/journal.pone.0075697

**Published:** 2013-09-25

**Authors:** Roger Kenneth Whealands Smith, Natalie Jayne Werling, Stephanie Georgina Dakin, Rafiqul Alam, Allen E. Goodship, Jayesh Dudhia

**Affiliations:** 1 Department of Clinical Sciences and Services, the Royal Veterinary College, University of London, Hatfield, United Kingdom; 2 Department of Biotherapeutics, National Institute for Biological Standards and Control, South Mimms, United Kingdom; 3 Institute of Orthopaedics & Musculo-Skeletal Science, University College London, Stanmore, United Kingdom; University of Liverpool, United Kingdom

## Abstract

Tendon injuries are a common age-related degenerative condition where current treatment strategies fail to restore functionality and normal quality of life. This disease also occurs naturally in horses, with many similarities to human tendinopathy making it an ideal large animal model for human disease. Regenerative approaches are increasingly used to improve outcome involving mesenchymal stem cells (MSCs), supported by clinical data where injection of autologous bone marrow derived MSCs (BM-MSCs) suspended in marrow supernatant into injured tendons has halved the re-injury rate in racehorses. We hypothesized that stem cell therapy induces a matrix more closely resembling normal tendon than the fibrous scar tissue formed by natural repair. Twelve horses with career-ending naturally-occurring superficial digital flexor tendon injury were allocated randomly to treatment and control groups. 1X10^7^ autologous BM-MSCs suspended in 2 ml of marrow supernatant were implanted into the damaged tendon of the treated group. The control group received the same volume of saline. Following a 6 month exercise programme horses were euthanized and tendons assessed for structural stiffness by non-destructive mechanical testing and for morphological and molecular composition.

BM-MSC treated tendons exhibited statistically significant improvements in key parameters compared to saline-injected control tendons towards that of normal tendons and those in the contralateral limbs. Specifically, treated tendons had lower structural stiffness (p<0.05) although no significant difference in calculated modulus of elasticity, lower (improved) histological scoring of organisation (p<0.003) and crimp pattern (p<0.05), lower cellularity (p<0.007), DNA content (p<0.05), vascularity (p<0.03), water content (p<0.05), GAG content (p<0.05), and MMP-13 activity (p<0.02).

Treatment with autologous MSCs in marrow supernatant therefore provides significant benefits compared to untreated tendon repair in enhancing normalisation of biomechanical, morphological, and compositional parameters. These data in natural disease, with no adverse findings, support the use of this treatment for human tendon injuries.

## Introduction

Tendon disease is a common age-related degenerative condition where current treatment strategies frequently deliver ineffective results in terms of functionality which impacts on quality of life, frequently with a recurrence of injury. Tendon disorders are a major direct and indirect financial burden on the NHS with more than 85,000 patients presenting to GPs each year with symptomatic Achilles tendinopathy [1] and 50,735 operations on tendon disorders being performed on the NHS in 2005/6 [2]. There are a multitude of therapies, ranging from surgical repair, rest alone and physiotherapy to corticosteroid injections, extracorporeal shockwave therapy, platelet rich plasma injections and eccentric loading exercises. However, apart from eccentric exercises for Achilles tendinopathy, none have been shown to be any more effective than placebo [3] and there is a need for improved non-surgical treatments.

Equine and human tendinopathy have many similarities in their pathophysiology [4]. Thus, both the superficial digital flexor tendon (SDFT) in the horse and the Achilles tendon in the human have similar structure and function, being elastic weight-bearing tendons having an energy-storing capacity for efficient locomotion. Both appear to accumulate degenerate change with age and exercise, which precede a high frequency of overstrain injury. Overstrain injury of the SDFT in the equine athlete remains devastating and often career ending. Epidemiological studies have demonstrated that these injuries account for 46% of all limb injuries on the racecourse and affect 24% of racehorses in training [5,6].

Tendons heal slowly with fibrosis following the initial inflammation leading to the formation of disorganised scar tissue. While the initial injury causes a reduction in structural stiffness, fibrosis frequently results in the tendon as a structure being becoming stiffer than normal tendon in the chronic stages [7,8]. This increased stiffness compromises the essential function of the SDFT as an elastic energy store for efficient locomotion [9] and results in a horse with compromised locomotor function and prone to re-injury [10]. An optimal treatment should, therefore, aim to restore normal structure and function. Consequently, there has been considerable interest in the potential therapeutic benefits of mesenchymal stem cells (MSCs) in regeneration of functional tendon and ligament. These cells reside in small numbers, within niches in all mesenchymal tissues, usually closely associated with blood vessels and are capable of differentiating into a number of different cell types [11,12]. As in other species, the ability of MSCs derived from both bone marrow and fat to differentiate into one [13] or more lineages has also been demonstrated for equine MSCs [14-19]. These cells may regenerate tendon tissue by differentiation into tendon cells or by modulating the inflammatory response to injury and consequent repair process [20,21].

Bone marrow (BM)-derived MSCs have been used in a large number of experimental laboratory animal models of acute tendon transection and have demonstrated continued viability and significantly improved outcomes over controls [22-27] supporting the translation of the technology into clinical use. In horses, Herthel [28] reported success with the direct injection of BM into damaged suspensory ligaments. However, injection of large volumes of BM (30-50 ml) causes further disruption of the healing tendon; additionally aspirated BM contains very few MSCs (~1 in 10^4^ nucleated cells) [18], bone spicules and fat cells which may be deleterious to tendon healing. Furthermore, there was no assessment as to whether functional tendon or scar tissue is formed after this treatment.

In an attempt to avoid the above concerns, Smith et al. [29] demonstrated the feasibility of *in vitro* isolation and expansion of equine BM-derived MSCs, with re-implantation of large numbers of autologous MSCs suspended in bone marrow supernatant into damaged equine SDFTs. The MSCs were combined with bone marrow supernatant so that the preparation would be completely autologous and also because bone marrow supernatant had been shown to provide a significant cellular anabolic stimulus [30,31]. Since this original publication, the technique has been widely adopted in many countries for the treatment of tendon (and ligament) overstrain injuries in horses. Experimental studies have been conducted, using collagenase tendon injury models in large animal species, which have demonstrated significantly improved outcomes with MSCs compared to saline injected controls [32,33]. In contrast, a different model based on acute mechanical disruption [34] failed to show the benefit of implanted stem cells over controls of BM supernatant [35], although this study only evaluated ultrastructure at one early (12 week) time-point with no mechanical or functional evaluation.

However, experimental models using induced acute injuries have limitations as they do not reflect all the features of clinical disease which commonly has a preceding phase of age-related degeneration [4]. In contrast, an adequately powered large clinical study of SDFT injuries in racehorses treated with autologous MSCs in bone marrow supernatant [36] demonstrated significantly reduced re-injury rates compared to two published case series of horses which had undergone a variety of other treatments [10,37]. However, there was no contemporaneous control population and no indication of a mechanism for the action of implanted MSCs on the healing of the damaged tendon matrix. Therefore, this study was initiated to explore the hypothesis that implantation of autologous BM-MSCs suspended in marrow supernatant into the site of injury in naturally injured SDFTs will induce a tissue more closely resembling normal tendon matrix than the fibrous scar tissue formed subsequent to natural repair in saline injected controls.

## Results

**Figure 1:**
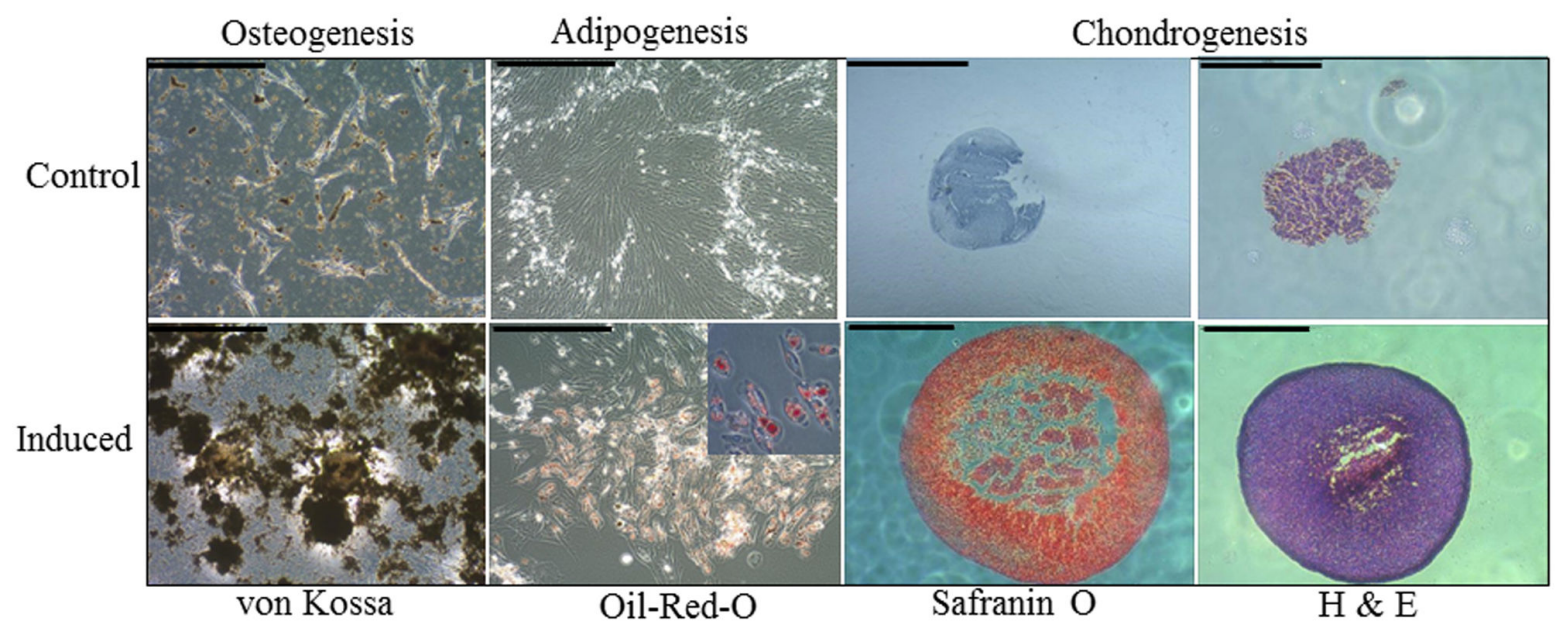
Trilineage differentiation of putative bone marrow MSCs. Representative microscopic images of von Kossa and Oil-Red-O staining to confirm osteogenic and adipogenic differentiation of plastic adherent bone marrow derived cell populations (scale bars 100 µm). A higher magnification inset is shown in the adipogenesis image to demonstrate the presence of intracellular lipid droplets. Chondrogenic differentiation was confirmed by the formation of dense cell pellets (haematoxylin and eosin staining) that were positive for Safranin O (orange-red stain) (scale bars 1 mm). All respective controls (non-induced cultures) are shown in the upper row panels.

There was no significant difference in the average ages of each group; 7±1.8 years for the stem cell group compared to 8.5±4.1years for the control group. The average interval between injury and treatment was also not statistically significantly different, with 49.3±14.6 days for the treated group and 54.8±10.0 days for the control group (Table 1). Analysis of the characteristics of the MSCs showed that there was no significant difference between cells prepared from the stem cell and saline groups, with an average population doubling time of 44.3±4.9 h and 47.5±4.2 h (Table 1). Both groups exhibited a similar clonogenic profile (Table 1). The MSCs from horses for which this assay was performed all showed trilineage (osteogenic, adipogenic and lipogenic) differentiation, and is summarised in Table 1. At 24 days in osteogenic media, the spindle-shaped cell morphology changed to dense stellate shaped clusters on which calcium deposition occurred as visualised by von Kossa staining (Figure 1). The control MSCs (non-induced medium) formed few or no stellate clusters and had little calcium deposition although small amounts of spontaneous mineral deposition was occasionally noted in some cultures. MSCs in adipogenic inducing media altered their morphology from spindle-shaped towards a more rounded cell shape with positive intracellular staining for Oil-Red-O. Control cells in non-inducing medium remained as a spindle-shaped monolayer with no intracellular lipid staining. Chondrogenic media induced the formation of macroscopically visible spherical colonies which increased in size over the 24 day culture period. These cell pellets stained positively with Safranin O for sulphated glycosaminoglycan moieties of proteoglycans indicating significant production and deposition of sulphated proteoglycans typical of cartilage. Central areas of necrosis were observed in large pellets, presumably due to limiting nutrient diffusion. Non-induced control cell pellets remained unchanged or decreased in size and in some cases were no longer visible at the end of the culture period. Where control pellets could be recovered, these showed poor staining for proteoglycans. There was intra-horse and inter-horse variability for each assay but in general osteogenesis was good across all horses whereas lipogenesis varied from positive to moderate (two horses). Chondrogenesis measured by pellet diameter was consistent in all horses albeit with some variability in the intensity for proteoglycan staining.

**Table 1:** Details of the horses in each treatment group and characteristics of equine bone marrow derived stem cells.

**Horse number and treatment group**	**Age (years**)	**Limb affected**	**Injury to implantation interval (days**)	**Analysis site (maximum injury zone**)	**PDT (hours**)	**CFU assay**	**Oestogenic potential**	**Chondrogenic potential**	**Lipogenic potential**
**Stem cell**									
1	6	RF	38	4	45	1.3	Positive	Moderate	Moderate
2	9	LF	75	4	38	1.1	Positive	Positive	Moderate
3	4	LF	33	4	44	0.8	Positive	Moderate	Positive
4	8	RF	47	4	50	1.0	Moderate	Positive	Poor
5	8	RF	51	3	nd	nd	nd	nd	nd
6	7	LF	52	3	nd	nd	nd	nd	nd
**Mean**	**7.0**		**49.3**		**44.3**	**1.1**			
**SD±**	**1.8**		**14.6**		**4.9**	**0.2**			
**Saline**									
1	15	RF	66	4	48	1.1	Positive	Moderate	Positive
2	11	LF	63	4	nd	nd	nd	nd	nd
3	9	LF	53	3	46	0.8	Positive	Positive	Positive
4	5	RF	49	3	53	0.6	Positive	Moderate	Poor
5	7	LF	39	4	nd	nd	nd	nd	nd
6	4	LF	59	4	43	0.9	Positive	Positive	Moderate
**Mean**	**8.5**		**54.8**		**47.5**	**0.9**			
**SD±**	**4.1**		**10.0**		**4.20**	**0.21**			

There were no significant differences between stem cell and saline groups for age, injury to implantation interval, CFU or PDT. LF, left forelimb; RF, right forelimb; CFU, colony forming unit; PDT, population doubling time; SD±, standard deviation; nd, not determined.

### In vivo analysis

**Figure 2:**
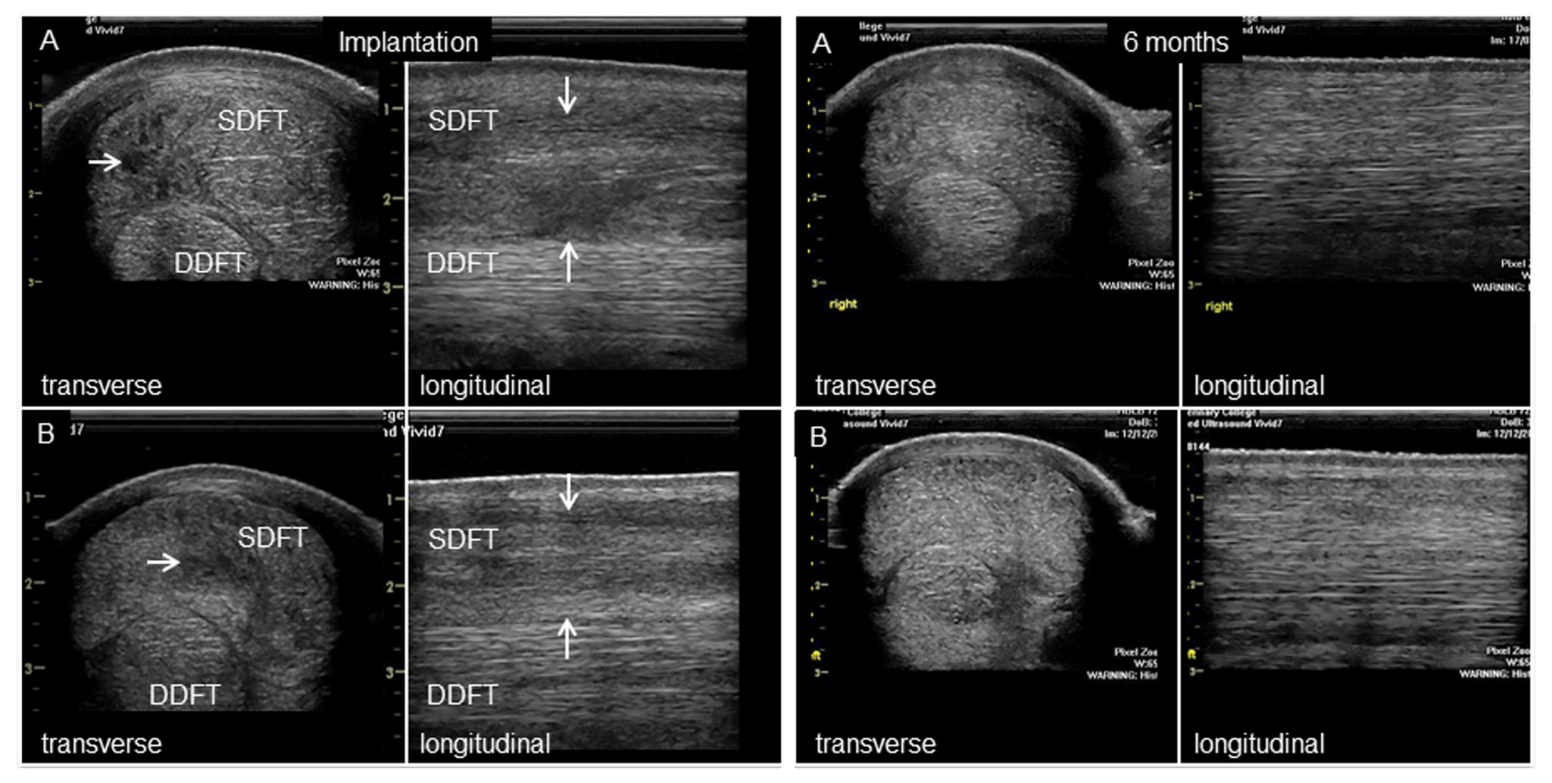
Ultrasonography of the superficial digital flexor tendon. Representative ultrasonographic appearance of (A) stem cell injected horses and (B) control (saline-injected) horses at the time of injection and at the conclusion of the study (6 months). Both transverse and longitudinal images are shown to illustrate the injury which is visible as a hypo-echoic area (arrows) denoting the loss of extracellular matrix, which is not present at 6 months. The linear organization of the healed tendon is apparent in the longitudinal images at 6 months.

All horses had moderate to marked ultrasonographic changes in the tendons (Figure 2). Most had generalised lesions with mixed central hypoechogenicity. Only three horses (2 stem cell and 1 saline control) had more focal hypoechoic core lesions. In all cases the contralateral limb had either no significant abnormalities on ultrasound (8/13 horses) or milder pathology (5/13 horses). No horses exhibited adverse effects after the intra-tendinous injections and all horses underwent the same post-injection exercise programme on the horse-walker (walking and trotting only) without lameness in 12 horses. One horse treated with stem cells was eliminated in further analysis due to sudden onset lameness during the rehabilitation phase of the study, which was associated with recurrent injury of a very severe tendon injury in the treated limb.

**Figure 3:**
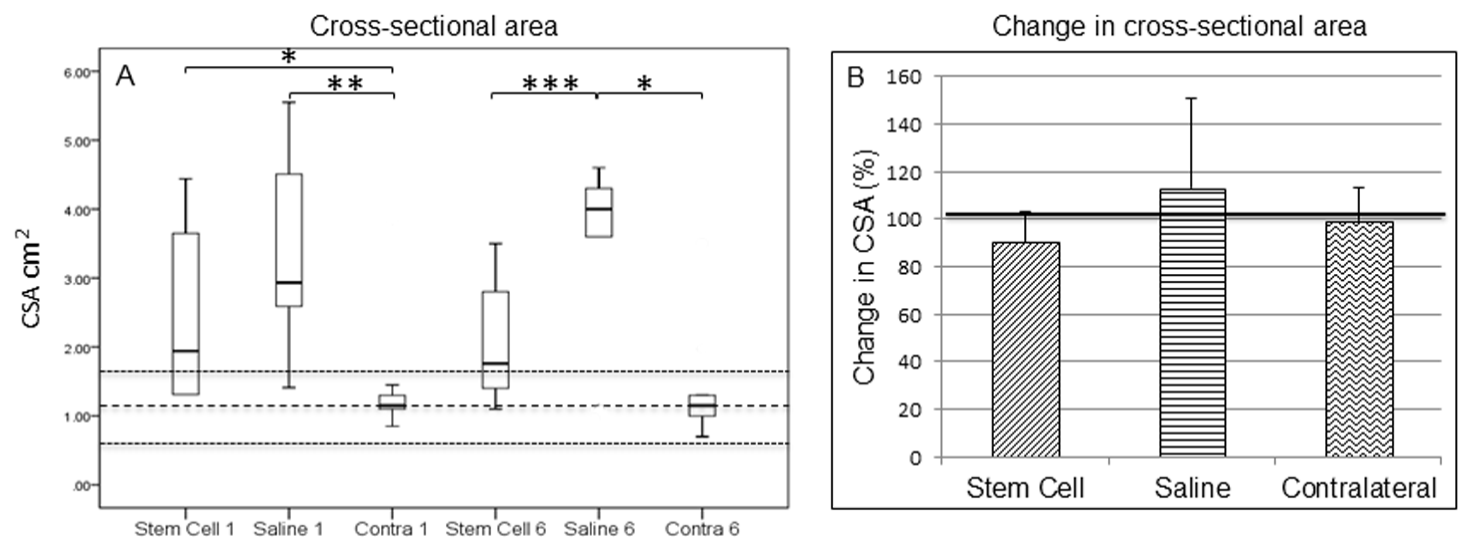
Cross-sectional area of the superficial digital flexor tendon. (A) Cross-sectional areas were measured ultrasonographically at the time of implantation (Stem Cell 1, Saline 1 and Contra 1) and at 6 months (Stem Cell 6, Saline 6 and Contra 6). (B) Relative change in cross-sectional area between groups. The Wilcoxon Signed-Rank test showed that there was no difference in cross-sectional area between 1 and 6 months for either the stem cell treated group or the saline group, or between the contralateral limbs in each group. Contralateral limbs were therefore combined from both groups for statistical analysis. The values for normal horses are shown as mean (thick dashed line) with two standard deviations (thin dashed lines). * p = 0.006, ** p= 0.003, ***p<0.045.

Cross-sectional area of the affected SDFT at the time of treatment (cell or saline injection) was larger than the normal cross-sectional area, consistent with injury [6], but was not significantly different between the groups (Figure 3A). After 6 months, however, there was no significant difference in the cross-sectional area in the stem cell treated group compared to the contralateral tendons, however the cross-sectional area in the saline control group remained significantly larger compared to the contralateral (p=0.006) and to stem cell treated (p=0.045) tendons. This was supported by the analysis of the change in cross-sectional area as a percentage of the cross-sectional area at the time of implantation, which showed that the treated group had a reduction in area while the control group increased although this was not statistically significant (Figure 3B). The cross-sectional area of the SDFT in the contralateral limbs was smaller than the affected tendons and inside the normal range, confirming the mild nature of pathology in the contralateral limbs. There was no statistically significant change in the cross-sectional area of the untreated contralateral limbs over the period of the rehabilitation. As there was no significant difference in the contralateral limbs between groups, the data for the contralateral limbs were pooled for the analysis.

### In vitro analysis

The hydration of the SDFT at the injury site of the treated group (65.63±3.78%) was not significantly different to the contralateral SDFT (62.73±2.2%) but both were significantly different to the saline treated control group (69.55±3.6%), (p=0.04 compared to the stem cell group; p=0.01 compared to the contralateral SDFT).

**Figure 4:**
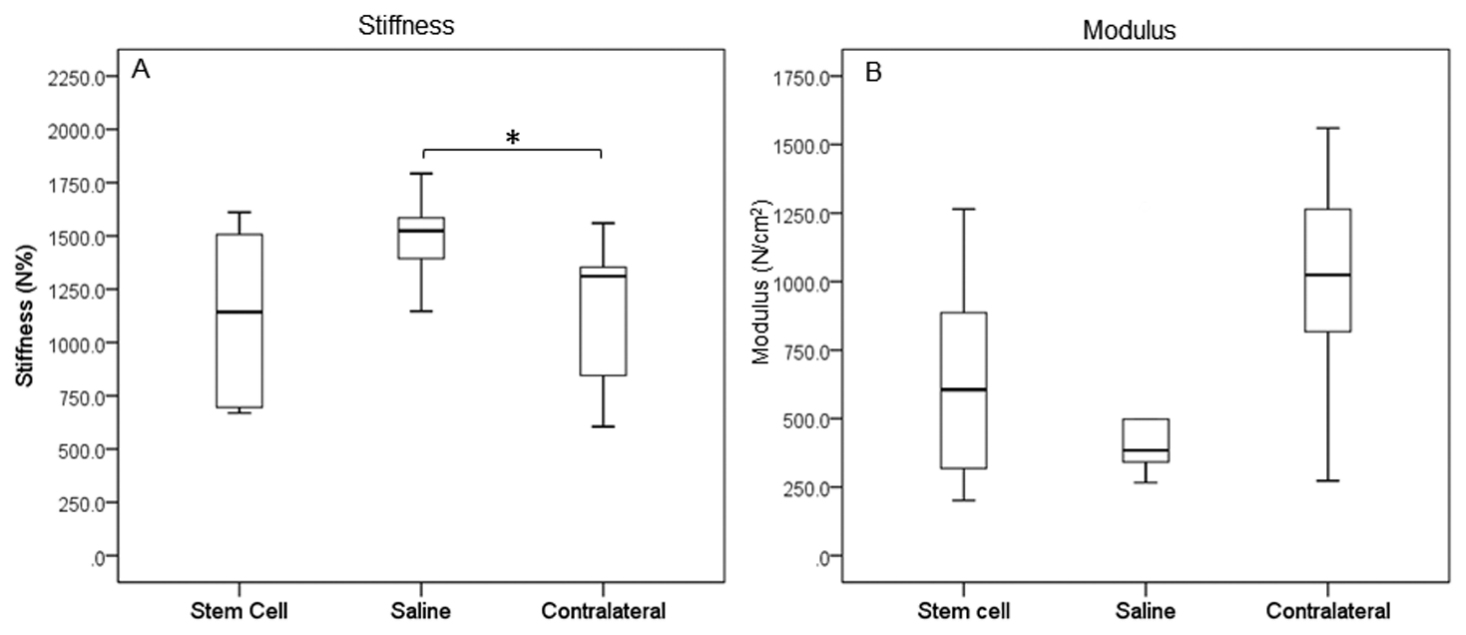
Biomechanical properties of tendons. SDFTs were subjected to mechanical testing within 2 h of post euthanasia to determine (A) structural stiffness and (B) material stiffness (modulus), which was calculated by expressing the ratio of the structural stiffness to the ultrasonographically-determined cross-sectional area. Structural stiffness was significantly reduced in stem cell treated tendons and closer to that of the uninjured (contralateral) tendons. * p=0.015.

A similar pattern was seen for tendon stiffness with the treated SDFT having significantly lower overall tendon structural stiffness than the control SDFT (by ~25%; p=0.015) and was similar to the stiffness of the contralateral tendon (2.2% different) (Figure 4). When a measure of the modulus of elasticity (tissue material property) was calculated using the cross-sectional area measured ultrasonographically at the maximum injury zone immediately prior to euthanasia, there was a higher modulus for the treated tendons compared to controls, although this was not statistically significant (Figure 4). In both cases, the modulus was not significantly different to the contralateral tendons.

**Figure 5:**
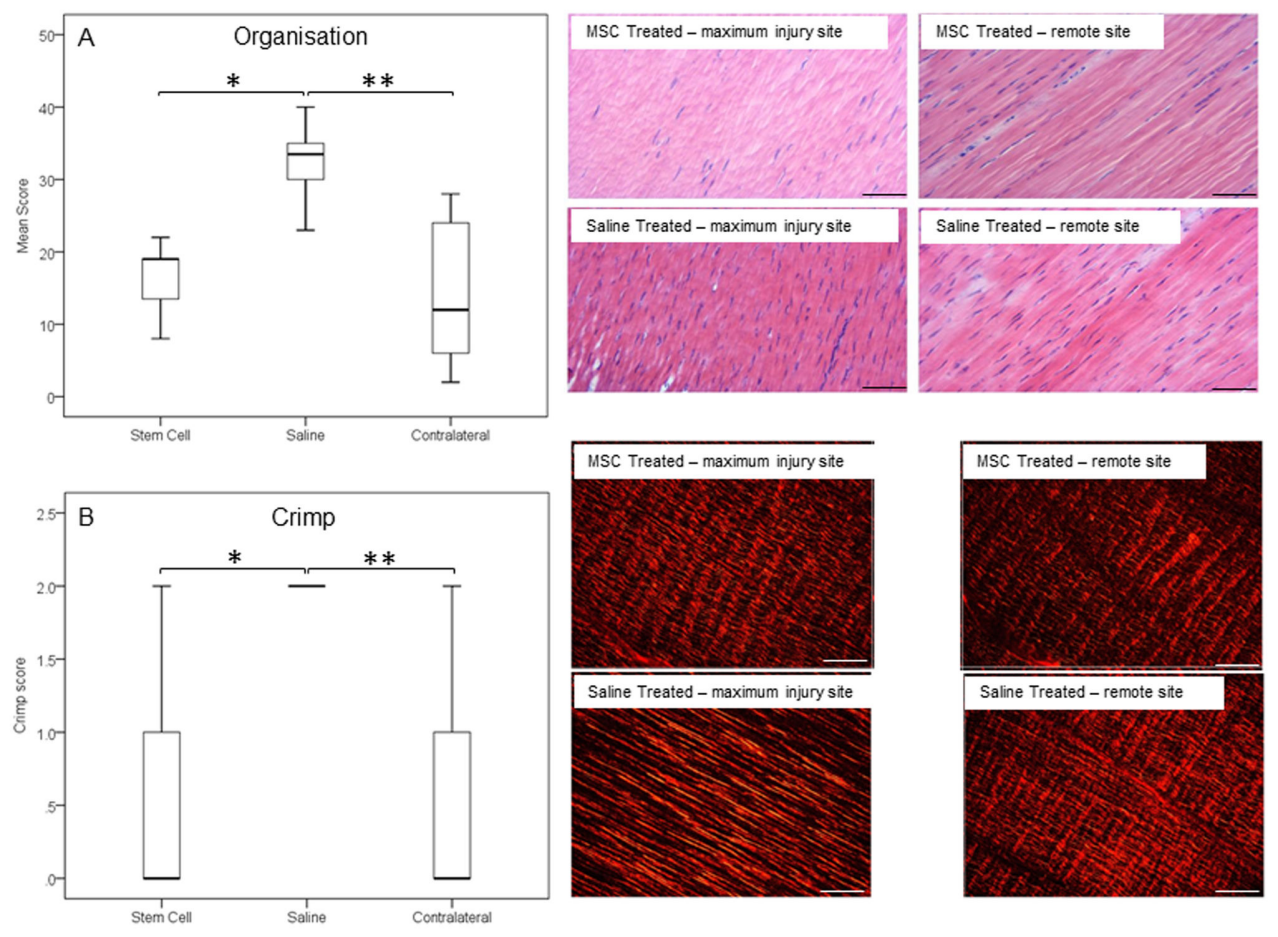
Morphological parameters of tendons. (A) Organisation and (B) crimp as assessed by blinded scores were taken from two separate histological sections for each tendon. Representative micrographs of haematoxylin and eosin stained histological SDFT sections from the treated and remote (less injured) sites in both MSC-treated and saline-treated groups are shown in the panels on the right. Normal tendon has a score of zero and the higher the score the more disrupted the tendon matrix and less crimp pattern, respectively. (A) * p = 0.003; ** p < 0.002; (B) * p = 0.046; ** p < 0.0001 (scale bars 50 µm).

**Figure 6:**
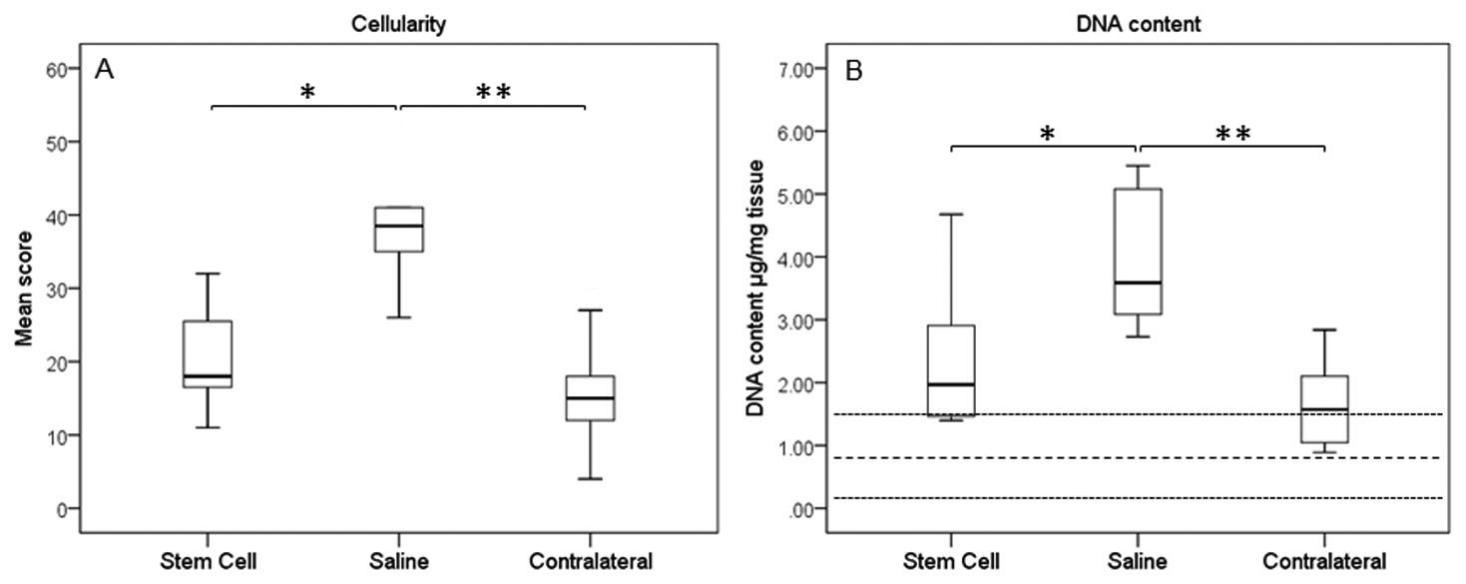
Cellularity of tendons. Both (A) cellularity scores and (B) DNA content demonstrated reduced cellularity in MSC-treated tendons. The values for normal horses are shown as mean (thick dashed line) with two standard deviations (thin dashed lines). (A) * p=0.007; ** p=0.001; (B) * p=0.037; ** p<0.001.

**Figure 7:**
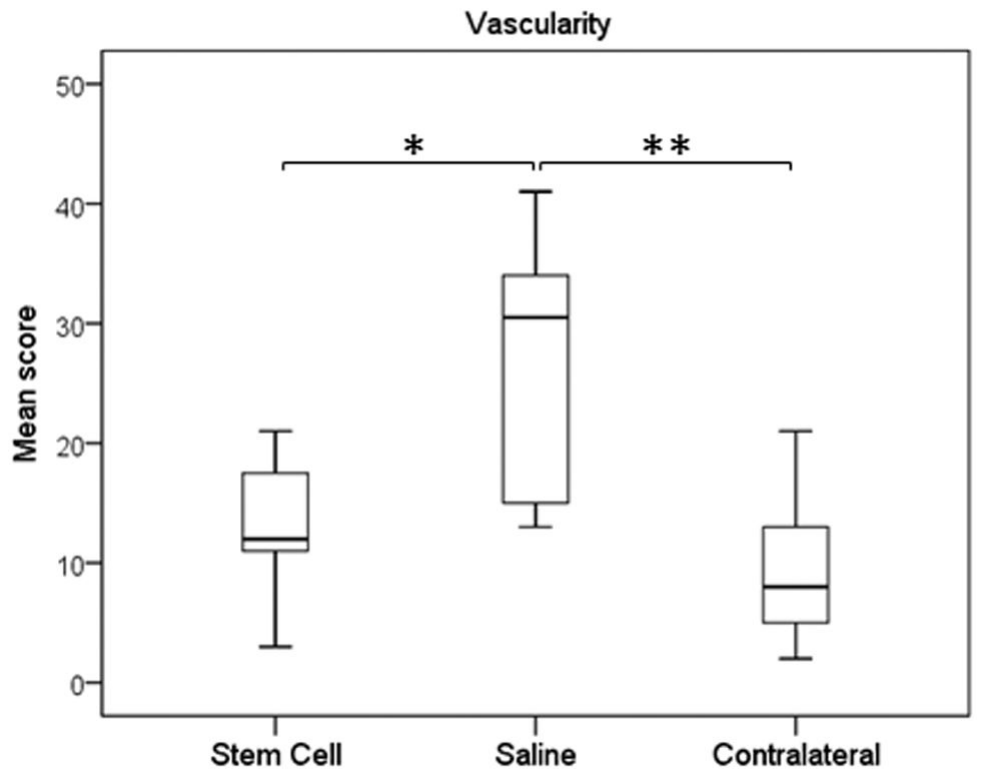
Vascularity of tendons. There was reduced vascularity in the MSC-treated tendons, similar to normal tendon, compared to saline-injected controls. * p = 0.026; ** p = 0.004.

Histologically, treated tendons had lower (indicating improved) organisational scores at the injured site compared to the control group, which were also significantly different to the contralateral tendons (Figure 5A; p=0.003 and p=0.002, respectively). The contralateral tendons had a score of 14.2±9.5 and so were not histologically normal reflecting the high frequency of bilateral (but milder) disease and there was no significant difference in scores between the contralateral limbs of both groups. This improved histological score in the treated tendons was supported by similar significant differences in the crimp scoring from polarised light microscopy (Figure 5B). It was also evident that the MSC-treated group had low cellularity scores (Figure 6; p=0.007), which again were comparable to remote, less injured sites. The lower cellularity was supported by lower DNA content (Figure 6; p=0.037). The treated group had significantly lower vascularity compared to the controls, which was similar to the contralateral limbs (Figure 7, p=0.026).

**Figure 8:**
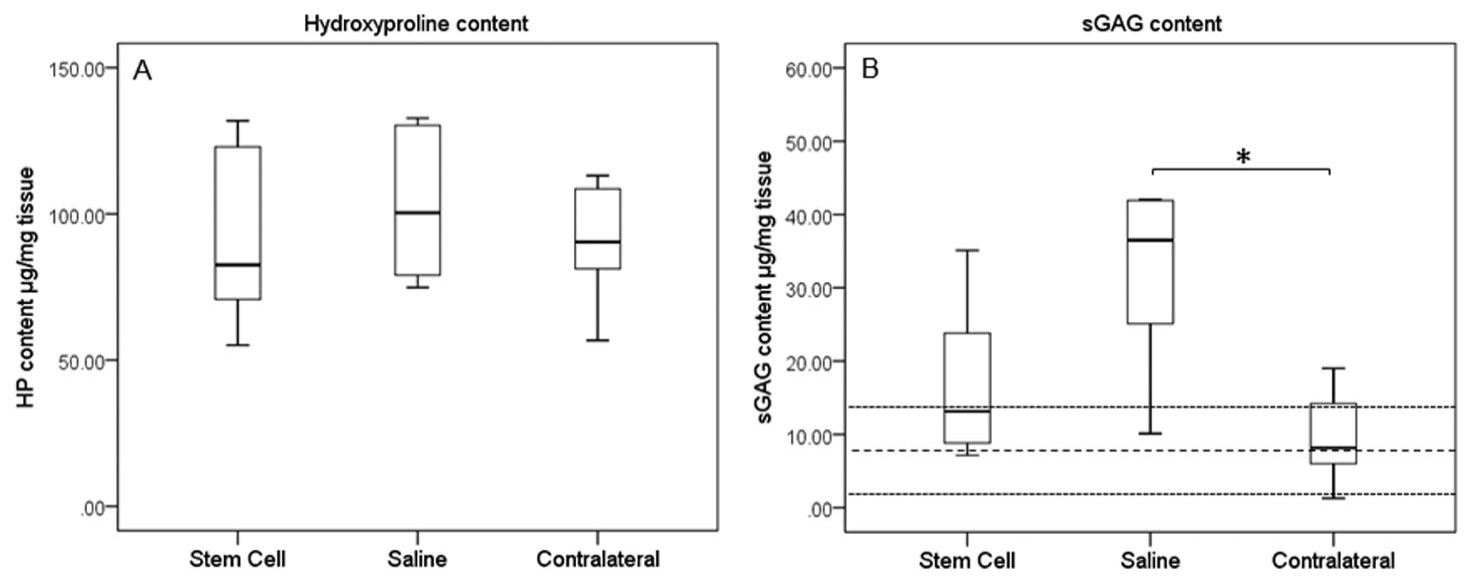
Compositional analysis of tendons. Concentrations for (A) hydroxyproline and (B) sGAGs are shown. The values for normal horses are shown as mean (thick dashed line) with two standard deviations (thin dashed lines). Note that the sGAG content of MSC-treated tendons was closer to the normal range and not significantly different from the relatively uninjured contralateral limbs (p = 0.24). * p = 0.05.

**Figure 9:**
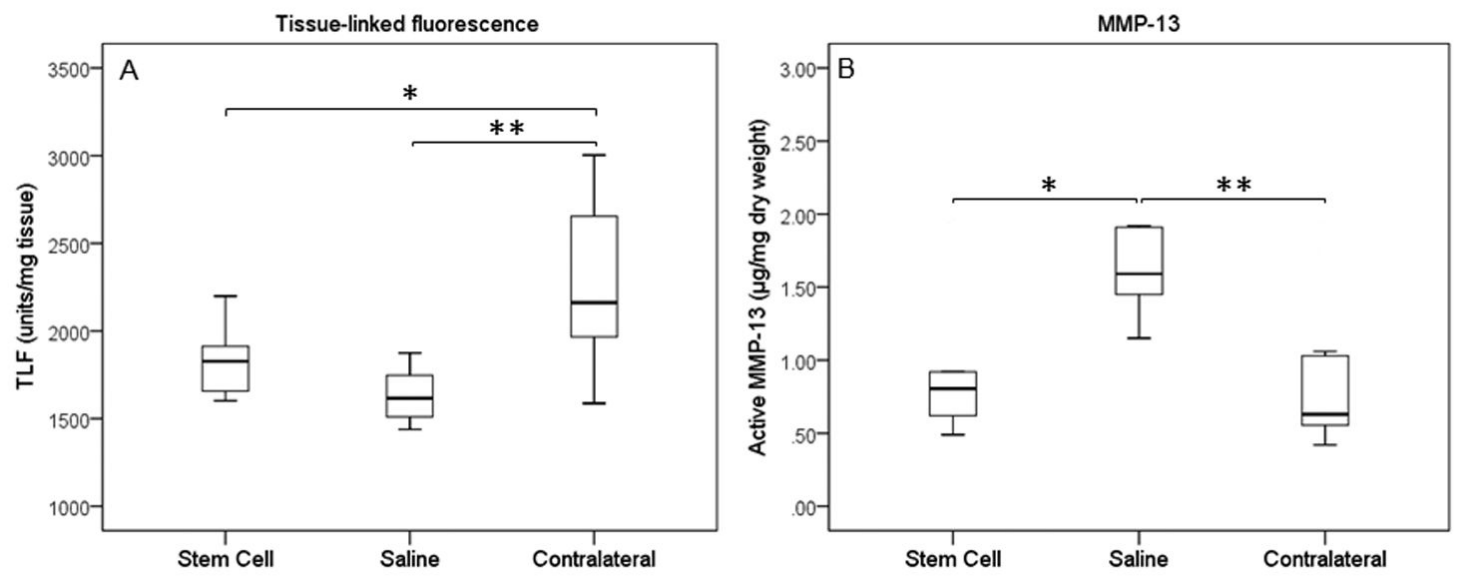
Collagen re-modelling in injured and stem cells treated tendons. (A) Tissue-linked fluorescence and (B) MMP-13 activity. The increased MMP-13 activity seen in the saline-treated group matched the lower tissue-linked fluorescence suggesting greater remodelling of the collagen matrix in the saline-treated compared to the MSC-treated tendons. (A) * p = 0.039; ** p = 0.003; (B) * p = 0.045; ** p = 0.015.

Total collagen (as hydroxyproline) content in MSC-treated SDFTs was unaltered between groups (Figure 8), while sGAG content was reduced and closer to the contralateral tendon, which was in the normal range published previously for normal tendons [38]. The sGAG content of the control group remained significantly elevated (p=0.005). There was no significant difference in the tissue-linked fluorescence between treatment and control groups although both were significantly lower than the contralateral limb (p=0.039 for treated and p=0.003 for controls) (Figure 9), which suggested similar amounts of new collagen in the injured limbs. A higher remodelling rate, exemplified by MMP-13 activity, was also demonstrated for the control group compared to the treated group (p=0.045) and contralateral limbs (p=0.015) (Figure 9).

## Discussion

The data presented here have supported the hypothesis that MSC implantation with BM supernatant results in a tissue more like normal tendon matrix rather than the fibrous scar tissue formed after natural inflammation and repair. For most parameters, the values for the treated group were closer to the contralateral, relatively uninjured, tendon than the control group and closer to the normal values available for some of the compositional parameters. This was further supported by significant differences in the organisational scores between injured and remote, less injured sites.

Many experimental models of induced acute tendon injury in laboratory animal models report increased material properties of the tissue (ultimate tensile stress and elastic modulus) [23,27] associated with stem cell treatment. In this study we report the overall structural properties of the tendon, which do not take account of the cross-sectional area. We have demonstrated a reduction in structural stiffness, which confers improved functional outcome as it will improve elastic energy storage and therefore locomotor efficiency. In addition, the reduced stiffness of the healed area minimises the abrupt strain gradient at adjacent, relatively normal tendon tissue, which is hypothesised to result in the high frequency of re-injury when the horse returns to high level exercise, seen clinically as recurrent tearing of the tissue at these ‘transition sites’ [39]. When the structural stiffness was corrected for the cross-sectional area at the maximum injury zone to give an approximate value for the modulus of elasticity, the modulus of the stem cell treated horses was not significantly different from the contralateral tendons, while the saline treated tendons had a lower modulus, although not statistically different from the treated group.

In addition to the mechanical properties, both organisational and compositional analyses reflected the same trend to normalisation of tissue parameters towards that of the contralateral limb, which, while not completely normal on ultrasonography and histology, were closer to normal tissue, supported by DNA content and sGAGs being within the range of normal tendons. In an experimental model study, these biochemical parameters remained elevated compared to normal at the end of the study at 24 weeks [40] although in both studies it is possible that they may have continued to change or remained stable with further rehabilitation as the usual length of rehabilitation for equine SDFT tendinopathy is approximately 12 months prior to a return to full athleticism.

The mechanism of action of MSCs with marrow supernatant after implantation has not yet been clarified. It has been proposed that they act either by inducing a true regenerative effect or by an immunomodulatory response by modifying the inflammatory response and indirectly modulating the subsequent fibrous repair processes of the host cells. Inflammation is often cited to be absent in tendon injuries, however we have recently shown the presence of a robust inflammatory response in natural disease and the presence of activated macrophages in the damaged tissue which appear to switch to a pro-resolving phenotype with disease phase [41,42]. It is tempting to speculate that MSCs play a role in modulating the pro-resolution phenotype of activated macrophages however it is not possible to determine which of these two actions (regeneration or immunomodulation) had occurred in this study. There is still persistent abnormal organisation and composition, which suggests that the effect does not involve a fully regenerative mechanism. This is in keeping with the findings in experimental studies using surgical or chemical models of tendon injury in horses where there has been no effect on ultrastructure in a surgical model [35] and only significant improvements in organisational scores in a chemically induced model [32]. The limited effects seen in these studies may be due to the differences between experimental models and naturally occurring injury. The nature of the tissue into which the cells were deposited was a naturally occurring injury with a preceding age-related degeneration which is very different from that induced acutely in experimental models. There was also a markedly different interval between treatment and when the tissue was harvested and analysed. The experimental studies evaluated the tendons 6-12 weeks after MSC implantation, while in this study, the reparative tissue was analysed after 24 weeks (6 months), at a time when many horses would be progressing to higher-level training.

It was intriguing to observe a lower cell count in the tendons that had received cell treatment. However, it is known from other studies that a large proportion of the implanted cells are lost [43,44], and this loss appears to occur early after implantation [45]. As the dynamics of the implanted cells have been evaluated, and because we have shown labels used for long term tracking of cells alter the viability and behaviour of implanted cells (data not shown), additional horses were not recruited to track the fate of the implanted cells in the current study. However, the number of implanted cells persisting to 6 months would be expected to be low and the overall lower cell numbers could also reflect an immunomodulatory effect in reducing hyperplasia of the tendon and the subsequent amount of fibrosis.

The preparation of stem cells used in this study consisted of a combination of MSCs and bone marrow supernatant, the latter of which has been shown to exhibit anabolic properties acting on tenocytes [30]. The treatment used was that found to reduce re-injury in a large equine clinical cohort study [36]. In this study it was not possible to determine whether the effect seen was caused by the MSCs, the supernatant, or both. Although we did not measure the biochemical composition of the bone marrow supernatants, its composition is similar between horses [46] which suggests a minimal variability in its contribution to the repair process. It is possible that growth factors in the bone marrow [30,46,47] could contribute to the beneficial effects of the implanted stem cells which is an additional possible explanation for the lack of response seen in a previous acute induced experimental model [35], where the control horses received bone marrow supernatant only and no significant effects were seen on ultrastructure (collagen fibril diameter) alone. Further work is needed to determine whether both components are necessary and act synergistically.

The higher MMP-13 activity seen in the saline treated group is consistent with the lower tissue-linked fluorescence, indicative of greater collagen turnover. However, the difference in tissue-linked fluorescence, may have been influenced by the differences in ages between the two groups (saline group were older on average although not significantly) as intrinsic fluorescence of long-lived tissue components, such as collagen, increases linearly with age by the accumulation of age-related advanced glycation end-products [48]. When plotted against age, the injured limbs had lower tissue-linked fluorescence than the contralateral limbs (data not shown) reflecting their acute pathology and greater matrix remodelling with old collagen replaced with new. The small differences in tissue-linked fluorescence most likely reflect the mixture of tissues present within the analysed sample. Given the severity of the recent injury, and the possible presence of previous pathology and fibrosis, it would not be surprising to encounter a mixture of newly formed collagen, normal aged collagen and older collagen from previous injury.

The significant reduction in sGAG levels in the treated group compared to the controls, which was only just outside the normal range, was considered a positive outcome, again representative at least in part, of a regenerative effect or normalisation of the healed tendon tissue closer to that of normal tendon. GAG levels are consistently elevated with pathology in tendons across many injuries in many species [49-54] and most treatments that enhance fibrosis tend to increase sGAG levels. The current study did not include analysis of the proteoglycan degrading enzymes ADAMTS4 and ADAMTS5 to determine if the accumulation of sGAGs correlated with altered expression and/or activity of these enzymes. Increased proteoglycan deposition in tendon disease correlates with ADAMTS5 knockout [55] and with its reduced expression in human tendinopathy [56]. It is intriguing that in equine suspensory ligament desmitis, chondroprogenitor cells appear responsible for elevated aggrecan synthesis and its accumulation may arise due to the inactivation of ADAMSTS5 despite its increased expression [57]. The role of implanted MSCs in modulating proteoglycan synthesis and the activity of these enzymes during tendon healing requires further investigation. The absence of a significant change in hydroxyproline levels between groups is not surprising as this parameter does not distinguish between different collagens in normal and fibrotic tendons. A more detailed analysis of changes in the expression of specific collagens is required to address this.

### Conclusion

This study has demonstrated that the technique of MSC implantation with marrow supernatant is not only safe but also indicates significant benefits to the healing tendon in normalising biomechanical (reduced stiffness), histological (better organisation) and compositional parameters (lower GAG content) towards those levels in normal tendon, which could be considered surrogate indicators of regeneration. The findings provide evidence that the implantation of autologous MSCs has beneficial therapeutic effects on the healing of superficial digital flexor tendon over-strain injuries which supports the reduction in re-injury rate seen clinically with this treatment [36]. In addition, these data support the potential use of MSCs in bone marrow supernatant for treating human tendon injuries.

## Materials and Methods

### Ethical Statement and recruitment of horses

The study was carried out following informed consent from the owners for horses to be donated to this study and under approval from the Ethics and Welfare Committee of the Royal Veterinary College (URN 2013 1230R 2005) and under UK Home Office Licences. Thirteen Thoroughbred and Thoroughbred cross horses (all male castrates), aged between 5 and 15 years of age (7.8±3.0 years), and suffering career-ending severe superficial digital flexor tendonitis within the metacarpal region were recruited for this study (Table 1). The selection criteria were that each horse had a recent injury to the SDFT in the mid-metacarpal region of less than 2 months (average 30 days between injury and entry into the study). Additional requirements were that the paratenon of the SDFT had to be intact and no horse had received previous intra-tendinous injections, thereby reducing potential loss of cells after implantation. Horses could have received anti-inflammatory agents but all systemically administered drugs had been cleared by the time of implantation because of the interval between entry into the study and treatment. It was not possible to determine with confidence if a previous injury had been present, although, as the horses had suffered career-ending injury which rendered them uneconomic to attempt to return them to racing, the injuries were severe, in many cases completely disrupting the entire tendon matrix (Figure 2). Naturally-occurring tendinopathy frequently manifests with bilateral involvement (a comprehensive epidemiological study documented a 35% frequency of bilateral injuries [6]) although the contralateral limb usually has milder pathology or else was injured previously (e.g. chronic disease). For the horses recruited for this study, pathology in the contralateral limbs was recorded but the limbs were not treated and were recovered for comparative evaluation.

### Ultrasonography of forelimbs

The SDFTs were examined by ultrasonography of the palmer aspect of the forelimbs, performed with the horse in weight-bearing stance as described previously [6,58]. Briefly, the limbs were prepared by clipping the hair, cleaned with a surgical scrub and then spirit and acoustic coupling gel applied. Images were obtained with a 13 MHz linear transducer to generate serial on-incidence transverse and longitudinal images of the soft tissues throughout the palmar metacarpal region (sequentially labelled levels 1-7 [6]) and stored digitally. Scans were performed at admission into the trial (time 0), then at 1 month (time point for cell or saline injection), 3 and 6 months. Transverse images of the SDFT were used to obtain the cross-sectional area (CSA) at the maximal injury zone (MIZ) of the injured tendon and at the same level of the contralateral limb with the aid of image analysis software (Scion Image, Scion Corporation, USA). All scans were obtained by a single operator (R.K.W.S).

### MSC isolation, in vitro culture expansion and characterisation

Autologous BM was recovered from the sternum under standing sedation using a standardised protocol [36] and shipped to the laboratory (VetCell BioScience Ltd, Kingham, Oxfordshire, UK) using validated shipping containers for MSC recovery and expansion.

In addition to cell expansion for *in vivo* implantation, the MSCs were characterised for their ability to undergo tri-lineage differentiation, population doubling time (PDT) and colony forming unit-fibroblast (CFU-F) assays.

### Osteogenic differentiation

MSCs (between passage 2-4) were seeded at a density of 1 ×10^4^ cells/cm^2^ in 35 mm^2^ dishes and allowed to expand until about 60-70% confluent in D10 medium (Dulbecco’s modified Eagle’s medium (DMEM), supplemented with foetal bovine serum (10% v/v), 100 U/ml penicillin, and 100 U/ml streptomycin) (Sigma-Aldrich, UK). The D10 was replaced with osteogenic media [59] which consisted of D10 medium supplemented with 10^-6^ M dexamethasone, 10 mM β-glycerophosphate, 0.02 mM L-ascorbic acid-2-phosphate (all from Sigma-Aldrich, UK). Control cultures were cultured in D10 medium alone. Media were replaced every 3-4 days for a total of 24 days. To assess mineralization, monolayers were fixed in 4% formaldehyde then stained with the von Kossa technique [60] using silver nitrate (Sigma-Aldrich, UK).

### Adipogenic differentiation

Cells seeded and expanded as above were induced to adipogenic differentiation in D10 supplemented with 1% ITSS media supplement (insulin, transferrin, sodium selenite), 1 µM dexamethasone, 100 µM indomethacin, 500 µM 3-isobutyl-1-methyl xanthine (all from Sigma-Aldrich, UK). After 72 h the cells were cultured in adipogenic maintenance media which consisted of D10 medium supplemented with 1% ITSS for a further 3 days. Four cycles (16 days) of this regime were performed, following which the cells were maintained in adipogenic maintenance media for a further 7 days [61]. Control cells were grown in maintenance medium for the entire period of differentiation. Cells were then fixed with 4% formaldehyde prior to staining with fresh Oil-Red-O solution to assess lipid droplet accumulation [61].

### Chondrogenic differentiation

2.5 x 10^5^ cells were centrifuged at 500 ×*g* for 5 minutes in 15 ml polypropylene conical tubes (FACS tubes; BD Biosciences, UK) and the pellet cultured in 0.5 ml of serum-free chondrogenic medium supplemented with 10 ng/ml transforming growth factor-β3, 0.1 µM dexamethasone, 0.17 mM ascorbic acid-2-phosphate, 1 mM sodium pyruvate, 0.35 mM L-proline, 1% ITSS, 1.25 mg/ml BSA, 5.33 µg/ml linoleic acid (Sigma-Aldrich, UK). Control cell pellets were grown in growth media. Media was replaced every three days up to 24 days after which the diameters of cell pellets were measured before fixing in 10% buffered formalin for 24 h and embedding in paraffin and sectioning. Sections (5–8 µm thick) were stained with 1% Safranin O and 0.02% Fast Green and counterstained with haematoxylin.

### Assessment of differentiation

The quality of the differentiation ability of the MSCs was visually assessed on the amount of coverage of respective stains for osteogenic and lipogenic differentiation. Thus, this was noted as negative if stained cells covered less than 10% of the dish; moderate if 10–50% and positive if greater. Chondrogenic differentiation was assessed as positive if pellet diameters grew to greater than 2 mm; moderate if 1-2 mm and poor if less than 1 mm. Proteoglycan deposition in cell pellets was confirmed by Safranin O staining of histological sections.

### CFU-F assays

Cells at low density (5 and 10 cells per cm^2^) were seeded in 35 mm dishes and cultured in D10 medium for 14 days at which time cell colonies were fixed in 4% formaldehyde for 10 mins. Colonies were visualised by staining with 0.5% crystal violet for 10 mins.

### Population doubling assays

Cells were seeded in triplicate at a density of 800 cells per cm^2^ and cultured for 3 days in D10 medium. Cells were then lifted by trypsin treatment and suspended in D10 for counting. Cells were re-seeded at the same density and cell counting repeated another 5 times. The population doubling time was calculated from the log_10_ of the ratio of the cell numbers at 36 h to the starting cell number.

### MSC implantation

10 x 10^6^ MSCs were suspended in 2 ml of citrated BM supernatant which was collected at the same time as the other BM samples in sodium citrate anticoagulant, centrifuged (1,500 x *g*) to remove cells and particulate material, and stored at -20 °C until required. The control group received 2 ml of isotonic saline (Dechra Veterinary Products, UK). For all horses, injections were performed on the most severely affected limb 3-4 weeks after bone marrow aspiration. A 2 inch 19G needle was guided under ultrasound guidance into the middle of the lesion (maximum injury zone) at 2-4 sites to ensure adequate spread throughout the lesion (as assessed ultrasonographically). The limb was then immediately bandaged and a single peri-operative dose of intra-muscular antibiotics (Norocillin, Norbrook Laboratories (GB) Ltd, Corby, UK) administered.

### Post-implantation exercise regime

The horses were box-rested with the limb bandaged for 7 days after which the horses began a standardised ascending exercise rehabilitation regimen of walking (increasing from 10-45 mins over the first 3 months) and then combined with trotting (increasing from 5-20 mins) in the second 3 months using a horse-walker. Horses were trotted in a straight line at the time of BM aspiration, pre-implantation and 1, 3 and 6 months after implantation to assess for obvious lameness (tendinopathy of the SDFT characteristically only causes significant lameness during the acute inflammatory stages (typically 1-2 weeks). At the same time, the palmar aspect of the metacarpus was evaluated ultrasonographically using standard transverse and longitudinal images of the SDFT as detailed above.

### Tendon analysis

Horses were euthanized after 6 months post-implantation and tendons were harvested from both forelimbs from each horse and analysed as follows

### Mechanical properties

The SDFT was removed in its entirety from both forelimbs and mounted in freezer clamps within 2 h in a 50 kN electro-hydraulic material testing machine (Dartec HA50; Zwick Roell Ltd, UK; retrofitted with an Instron 8800 control system; Instron, High Wycombe, UK) to give an inter-clamp (gauge) length of between 120 and 250 mm consisting of the damaged area of the SDFT. Non-destructive testing was performed on the tendon with loading up to 4 kN (one quarter of the average failure load found in similar equine tendons) for 20 cycles (to allow for pre-conditioning) at 0.5 Hz. The stiffness was calculated from between 3 and 4kN on the last loading cycle where the points lay on a linear part of the force-deformation curve. The gradient of the best-fit line (R^2^>0.99 in all cases) was used as the stiffness (N/%).

After mechanical testing the SDFT was divided into seven 4 cm sections matching the extent of the ultrasonographic examination from proximal to the distal extent of the SDFT. Each 4 cm section was sub-divided into four 1 cm sections. Peripheral tissue was discarded before the samples were prepared for histology and compositional analysis.

### Histological examination

Tendon sections were prepared for histology as previously described [58]. Briefly, tendon sections were embedded in optimal cutting temperature compound (OCT, Sakura Tissue-Tek®, The Netherlands), and snap frozen in pre-chilled (-80 °C) n-hexane and stored at -80 °C. Serial sections (8–10 µm thickness) in the longitudinal plane were cut on a cryostat (Bright Instrument, UK) and mounted onto poly-L-lysine coated slides (VWR, UK) and stained with Harris’s haematoxylin and 1% alcoholic eosin (Sigma-Aldrich, UK). Sections were cleared in Histoclear before mounting in DPX mounting medium (BDH, Poole, UK). Stained sections were imaged at the same magnification (x 20 objective lens; Olympus BX60 microscope) to obtain five images each for the peripheral and central (core) regions of the tendon. Two sections were imaged for each sample and evaluated in a blinded fashion for cellularity, vascularity and extracellular matrix organisation based on the system previously described [62,63] and summarised in Table 2. Scores from each image were combined for each variable to give a total score for the tendon for each horse.

**Table 2:** Scoring of histological sections to assess cellularity, vascularity and collagen fibre organisation.

**Cellularity score**	**Appearance**	**Description**
0	Normal	Presence of flattened cells in linear pattern between fibres
1	Slightly abnormal	Some rounded cells present, slight increase in cellularity
2	Abnormal	Many rounded cells present, obvious increase in cellularity
3	Markedly abnormal	Mostly rounded cells present, much higher numbers
**Vascularity score**	**Appearance**	**Description**
0	Normal	Presence of some vascular bundles parallel to collagen fibres
1	Slightly abnormal	Slight increase in number of vascular bundles
2	Abnormal	Increased number of vascular bundles
3	Markedly abnormal	Large increase in number of vascular bundles
**Organisation score**	**Appearance**	**Description**
0	Normal	Parallel collagen fibres of similar widths
1	Slightly abnormal	Some loss of fibre organisation, some loss of linearity
2	Abnormal	Moderate loss of fibre organisation, few linear regions
3	Markedly abnormal	Total loss of organisation, no linear fibres
**Crimp score**	**Appearance**	**Description**
0	Normal	Regular crimp angle and parallel spacing of fibres
1	Reduced	Irregular crimp angle and spacing
2	Absent	Poorly defined crimp angle or absent

Scoring was based on the system described by Movin et al (1997) [63].

For crimp analysis, cryosections were fixed in ice cold methanol (100%) for 5 minutes then washed twice in PBS. Sections were stained with 0.1% Direct Red 80 (Sigma Aldrich, UK) in saturated picric acid for 1 hour. After a 5 minute wash in 0.5% acetic acid, sections were dehydrated and mounted. Sections were imaged under polarised light (Olympus CX31-P microscope) and were scored in a blinded fashion for banding patterns indicative of crimp. A score of 0 indicated a normal banding pattern, a score of 1 indicated a reduced banding pattern, and a score of 2 indicated no banding (Table 2).

### Compositional analysis

One tendon section (1 cm section from each level) was freeze-dried and ground to a powder prior to analysis of tissue content. Water content was measured from the weight of the tissue section before and after freeze-drying.

Collagen content was determined by a measure of hydroxyproline content. Aliquots (10 mg) of ground tissue were suspended in papain digestion buffer (PBS containing 5 mM EDTA, 5 mM L-cysteine HCl and 125 µg/ml papain (all from Sigma-Aldrich, UK) and digested at 60 °C for 24 h and the solubilised collagen hydrolysed with 6 N HCl at 105 °C for 24 hours in sealed tubes. The aqueous phase was then removed by freeze-drying and the solid residue reconstituted in distilled water. Aliquots in triplicate were then assayed for hydroxyproline in a microtitre plate assay. Briefly, the sample was made to 35% isopropanol, 12 mM Chloramine T, 0.06 M sodium acetate and 0.05 M trisodium citrate and 4 mM citric acid. This was then incubated at 70 °C in 0.3 M 4-dimethylaminobenzaldehyde, 7% Perchloric acid and 40% isopropanol. The absorbance of the samples at 540 nm was measured after 20 mins. Absorbance values were quantified using a standard curve consisting of hydroxyproline (Sigma-Aldrich, UK) dilutions (50 to 0.78125 µg/ml) treated as above.

Glycosaminoglycan (sGAG) content of papain digested tissue was determined by the dimethylmethylene blue (DMMB) assay [64]. Standards were made from bovine trachea chondroitin sulphate (Sigma-Aldrich, UK). 10 µL aliquots of standards or media samples were added to a 96 well flat-bottomed plate in triplicate. 100 µL of DMMB (Sigma-Aldrich, UK) solution consisting of 46 µM DMMB, 0.2% methanol, 40 µM sodium chloride, 40 µM glycine and 10 mM HCl (pH 3.5) was then added to each well and the absorbance measured at 530 nm within 15 minutes on a spectrophotometer (Applied Biosystems UK). sGAG concentration of samples was derived from the standard curve expressed as µg/ml and subsequently adjusted to represent sGAG per mg of tissue.

### DNA and tissue linked fluorescence assays

DNA as a measure of tissue cellularity and tissue-linked fluorescence as a measure of the age of tissue components were determined on aliquots of papain digested tissue extracts prepared as described above. DNA was measured using bisBenzimide (Hoechst 33258, Sigma Aldrich, UK) and tissue-linked fluorescence was measured in the absence of bisBenzimide [62]. Samples were corrected for background fluorescence and the DNA concentration calculated from a standard curve prepared with calf thymus DNA (Sigma Aldrich, UK). DNA content of tendon samples was expressed as micrograms of DNA per mg of dry weight tissue and tissue linked fluorescence as arbitrary units per milligram of collagen (measured as hydroxyproline content).

### MMP-13 assay

A sample of freeze-dried powdered tendon was mixed with 100 volumes (w/v) of extraction buffer (50 mM Tris pH 7.5, 150 mM NaCl, 5 mM CaCl_2_, 1 µM ZnCl_2_, 0.01% Brij 35) at room temperature for 90 mins. Supernatants were recovered by centrifugation (20,000 x *g* for 10 mins at room temperature and frozen at -20 °C until further analysis. MMP-13 in the supernatants was measured by a fluorogenic substrate assay (Calbiochem, UK) according to manufacturer’s instructions. Briefly, 1 µl of fluorogenic substrate was added to 25 µl of tissue extract and 74 µl of assay buffer (50 mM HEPES, 200 mM NaCl, 1 mM CaCl_2_, 0.01% Brij 35) in a 96-well plate. Plates were incubated at 37 °C in the dark for 2 h and plates read at excitation wavelength 325 nm and emission wavelength 393 nm. Plates were incubated a further 6 h at 37 °C in the dark and measured every hour. A standard curve was constructed using human recombinant active MMP-13 (Calbiochem, UK). Standard and samples were assayed in duplicate and the results expressed as µg active MMP-13 per mg dry weight tissue.

### Statistical analysis

Histology scores and biochemical data were analysed by a non-parametric Mann-Whitney U-test as described for Movin scores of tendon by Maffulli et al. [65] Tendon cross-sectional areas were subjected by Kruskal-Wallis non-parametric one way ANOVA to identify differences between groups (stem cell treated, saline treated and contralateral) followed by a post-hoc Mann-Whitney test with Bonferroni correction. A detailed comparison of the contralateral limbs within and between the groups (stem cell or saline) using an independent samples or paired T-test (or their non-parametric equivalent) showed that there was no difference between the groups or with time (implantation time compared to 6 months). Therefore the results are shown with the contralateral limbs as a single group pooled from both treatment groups. The n values therefore are n = 6 each for stem cell and saline injection groups and n = 12 for the contralateral group. Significance level was set at 5% (p≤0.05). Data are expressed as mean ± standard deviation (SD).
